# Calcium intake is associated with decreased prevalence of periodontal disease in young Japanese women

**DOI:** 10.1186/1475-2891-13-109

**Published:** 2014-11-24

**Authors:** Keiko Tanaka, Yoshihiro Miyake, Hitomi Okubo, Takashi Hanioka, Satoshi Sasaki, Nobuyuki Miyatake, Masashi Arakawa

**Affiliations:** Department of Hygiene, Faculty of Medicine, Kagawa University, Miki, Kagawa 761-0793 Japan; Department of Public Health, Ehime University Graduate School of Medicine, Ehime, Japan; Department of Health Promotion, National Institute of Public Health, Saitama, Japan; Department of Preventive and Public Health Dentistry, Fukuoka Dental College, Fukuoka, Japan; Department of Social and Preventive Epidemiology, School of Public Health, The University of Tokyo, Tokyo, Japan; Course of Wellness, Graduate School of Tourism Sciences, University of the Ryukyus, Okinawa, Japan

**Keywords:** Calcium, Cross-sectional studies, Periodontal disease, Women

## Abstract

**Background:**

We investigated the relationships between calcium intake and the prevalence of periodontal disease.

**Methods:**

This cross-sectional study included 1162 women with a mean age of 31.5 years. Information on dietary factors was collected using a diet history questionnaire during pregnancy. Oral examinations were performed between one and twelve months postpartum. Periodontal disease was defined as positive if a woman had at least one tooth with a pocket depth of 4.0 mm or deeper. Adjustment was made for age, region of residence, smoking status, toothbrushing frequency, use of an interdental brush, household income, and education.

**Results:**

Compared with the lowest quartile of calcium intake, the highest quartile was significantly associated with a lower prevalence of periodontal disease; however, the inverse linear trend fell just short of the significance level: the adjusted odds ratio was 0.53 (95% confidence interval: 0.30–0.94, *P* for trend =0.07).

**Conclusions:**

Our findings suggest that higher calcium intake may be inversely associated with the prevalence of periodontal disease.

## Background

Periodontal disease is a chronic condition characterized by loss of tooth-supporting connective tissue and alveolar bone [1]. A complex relationship among bacterial, host, behavioral and environmental factors determines the development and progress of the disease [2]. As with various other chronic diseases, intake of foods and nutrients is considered an important factor, but epidemiological evidence on the relationship between diet and periodontal disease has been limited, and the results have been inconsistent [3, 4].

Calcium is the most abundant mineral in the human body. Although the majority of calcium in the body is in the structure of bones and teeth, the remaining calcium performs functions so essential to life that they take first priority over bone mineralization [5]. Calcium status in humans is likely to influence bone health, including alveolar bone health. Calcium is one of the relatively more widely studied nutrients in relation to periodontal disease [6–12]. A US cross-sectional study using data from the Third National Health and Nutrition Examination Survey observed a significant positive association between low dietary calcium intake and periodontal disease among young males and females (20 to 30 years of age) and older males (40 to 59 years of age) [8]. In older Danish adults, a higher intake of calcium from dairy products, but not from foods other than dairy products, was significantly inversely associated with the prevalence of periodontal disease [10]. In a longitudinal study among the elderly in Japan, an inverse dose–response relationship between serum calcium levels and periodontal disease was observed in smokers but not in non-smokers [11]. On the other hand, no association was observed between dietary calcium intake and periodontal disease in a case–control study of German adults [12]. Thus, the results on the association between calcium intake and periodontal disease have been inconsistent. Further evidence is needed to clarify the influence of calcium intake on periodontal disease.

In the present study, we assessed the relationship between calcium intake and the prevalence of periodontal disease among young Japanese women, using the data set of the Kyushu Okinawa Maternal and Child Health Study (KOMCHS).

## Methods

### Study population

**Figure 1:**
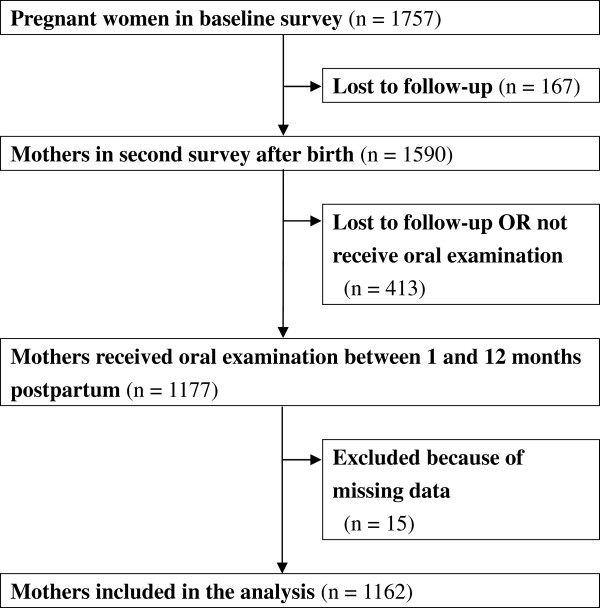
**Flowchart of the study population in the current analyses.**

The KOMCHS is an ongoing prospective prebirth cohort study that investigates risk and preventive factors for maternal and child health problems such as oral health and allergic disorders. Eligible subjects were those women who became pregnant in one of seven prefectures on Kyushu Island in southern Japan or Okinawa Prefecture between April 2007 and March 2008. At 423 obstetric hospitals, a set of leaflets explaining the KOMCHS, an application form to participate in the study, and a self-addressed and stamped return envelope were distributed to pregnant women, insofar as this was possible. Pregnant women who intended to participate in the KOMCHS returned the application form to the data management center. In the end, a total of 1757 pregnant women between the 5th and 39th week of pregnancy gave their written informed consent to participate and also completed the baseline survey. A flowchart depicting the study population included in our analysis is provided in Figure 1. The ethics committee of the Faculty of Medicine, Fukuoka University approved the KOMCHS. The STROBE (Strengthening the Reporting of Observational studies in Epidemiology) guidelines were followed.

### Outcome variable

Oral examinations to evaluate the condition of periodontal tissue between one and twelve months postpartum were performed by dental hygienists. Examination was performed under good natural light using a dental mirror and a Community Periodontal Index (CPI) probe (YDM Corp., Tokyo, Japan). Probing pocket depth (PPD) was determined with a CPI probe at six sites per tooth for six teeth: the right first molar, right first incisor, and left first molar in the maxilla and the right first molar, left first incisor, and left first molar in the mandible. When the target tooth was missing, the second molar in the same side or the first incisor in the opposite side was examined. The deepest PPD was recorded for each tooth. Periodontal disease was defined as positive if a woman had at least one tooth with a PPD of 4.0 mm or deeper.

### Exposure variables and covariates

In the baseline survey, each participant filled out a two-part questionnaire and mailed it to the data management center; all data were derived from a single questionnaire mailed directly to the participants. Research technicians completed missing or unclear data by telephone interview. The first part of the questionnaire elicited information on age, region of residence, smoking habit status, toothbrushing frequency, use of an interdental brush, household income, and educational level. The second part of the questionnaire was a semi-quantitative, comprehensive diet history questionnaire (DHQ) that assessed dietary habits during the preceding month [13, 14].

Estimates of daily intake of foods (total of 150 foods), energy, and selected nutrients were calculated using an ad hoc computer algorithm for the DHQ based on the Standard Tables of Food Composition in Japan [15]. Information on dietary supplements was not used due to the lack of a reliable composition table for dietary supplements in Japan. Also, only a small number of participants (6.1%) used calcium supplements at least once per week. According to a validation study of 92 females aged 31 to 69 years, Pearson’s correlation coefficient between the DHQ and 16-day weighted dietary records was 0.56 for calcium [16]. Energy-adjusted intake by the residual method was used for the analyses [17].

### Statistical analysis

Intake of calcium was categorized into quartiles on the basis of the distribution in 1162 subjects. Age, region of residence, smoking status, toothbrushing frequency, use of an interdental brush, household income, and educational level were selected *a priori* as potential confounding factors. Region of residence was classified into three categories (Fukuoka Prefecture, other than Fukuoka Prefecture on Kyushu Island, and Okinawa Prefecture), smoking status into two (never and ever), toothbrushing frequency into three (<2, 2, and ≥3 times/day), use of an interdental brush into two (no and yes), household income into three (<4,000,000, 4,000,000 - 5,999,999, and ≥6,000,000 yen/year), educational level into three (<13, 13–14, and ≥15 years). Age was used as a continuous variable.

Logistic regression analysis was performed to estimate crude odds ratios (ORs) and their confidence intervals (CIs) for periodontal disease in relation to calcium intake. Multiple logistic regression analysis was employed to adjust for potential confounding factors. Trend of association was assessed by a logistic regression model in which the median value in each quartile of calcium intake was assigned as the representative score. Two-sided *P* values less than 0.05 were considered statistically significant. All statistical analyses were performed using the SAS software package version 9.3 (SAS Institute, Inc., Cary, NC, USA). The statistical power calculation was performed using QUANTO version 1.2 [18].

## Results

The prevalence of periodontal disease among the 1162 women was 11.4%. The mean age of the participants was 31.5 years (Table 1). About 70% of women were never smokers. Toothbrushing two and three or more times per day was reported for 50.4% and 36.8% of participants, respectively. An interdental brush was used by about 46% of women. Mean daily total energy intake was 7396.0 kJ, and mean energy-adjusted intake of calcium was 500.2 mg.

**Table 1:** **Distribution of characteristics of 1162 women, KOMCHS, Japan**

Variable	Number (%) or mean ± SD
**Age (years)**	31.5 ± 4.2
**Region of residence**	
Fukuoka Prefecture	734 (63.2)
Other than Fukuoka Prefecture in Kyushu	321 (27.6)
Okinawa Prefecture	107 (9.2)
**Smoking status**	
Never	818 (70.4)
Ever	344 (29.6)
**Toothbrushing frequency (times/day)**	
< 2	149 (12.8)
2	586 (50.4)
≥ 3	427 (36.8)
**Use of an interdental brush**	
No	627 (54.0)
Yes	535 (46.0)
**Household income (yen/year)**	
< 4,000,000	374 (32.2)
4,000,000–5,999,999	418 (36.0)
≥ 6,000,000	370 (31.8)
**Educational level (years)**	
< 13	239 (20.6)
13–14	386 (33.2)
≥ 15	537 (46.2)
**Total energy intake (kJ/day)**	7396.0 ± 1955.5
Calcium intake (mg/day)*	500.2 ± 220.9

*Calcium intake was adjusted for total energy intake using the residual method.

The crude OR for periodontal disease in relation to every one-year increase in age was 1.04 (95% CI: 1.00–1.09) (Table 2). Compared with living in Fukuoka Prefecture, living in a prefecture in Kyushu other than Fukuoka Prefecture was associated with increased prevalence of periodontal disease. Compared with the lowest quartile of calcium intake, the highest quartile was significantly associated with a lower prevalence of periodontal disease. After adjustment for confounding factors under study, the inverse association was more evident: the adjusted OR between the extreme quartiles was 0.53 (95% CI: 0.30–0.94). The inverse linear trend between calcium intake and periodontal disease, however, fell just short of the significance level (*P* for linear trend =0.07).

**Table 2:** **Odds ratios and 95% confidence intervals for periodontal disease 1162 women, KOMCHS, Japan**

	Prevalence	Crude OR (95% CI)	***P***value	Adjusted OR (95% CI) ^†^	***P***value
**Age (years)**		1.04 (1.00, 1.09)	0.06	1.04 (0.99, 1.09)	0.09
**Region of residence**					
Fukuoka Prefecture	49/734 (6.7%)	1.00		1.00	
Other than Fukuoka Prefecture in Kyushu	80/321 (24.9%)	4.64 (3.17, 6.85)	<0.0001	4.84 (3.28, 7.24)	<0.0001
Okinawa Prefecture	3/107 (2.8%)	0.40 (0.10, 1.13)	0.13	0.41 (0.10, 1.14)	0.14
**Smoking status**					
Never	88/818 (10.8%)	1.00		1.00	
Ever	44/344 (12.8%)	1.22 (0.82, 1.78)	0.32	1.51 (0.99, 2.29)	0.05
**Toothbrushing frequency (times/day)**					
< 2	17/149 (11.4%)	1.00		1.00	
2	58/586 (9.9%)	0.85 (0.49, 1.55)	0.59	0.84 (0.46, 1.57)	0.56
≥ 3	57/427 (13.4%)	1.20 (0.69, 2.19)	0.54	1.05 (0.57, 2.01)	0.87
**Use of an interdental brush**					
No	66/627 (10.5%)	1.00		1.00	
Yes	66/535 (12.3%)	1.20 (0.83, 1.72)	0.33	1.14 (0.77, 1.69)	0.51
**Household income (yen/year)**					
< 4,000,000	42/374 (11.2%)	1.00		1.00	
4,000,000–5,999,999	42/418 (10.1%)	0.88 (0.56, 1.39)	0.59	0.88 (0.54, 1.43)	0.61
≥ 6,000,000	48/370 (13.0%)	1.18 (0.76, 1.84)	0.47	1.13 (0.69, 1.87)	0.63
**Educational level (years)**					
< 13	24/239 (10.0%)	1.00		1.00	
13–14	49/386 (12.7%)	1.30 (0.78, 2.22)	0.32	1.34 (0.78, 2.38)	0.30
≥ 15	59/537 (11.0%)	1.11 (0.68, 1.86)	0.69	1.17 (0.68, 2.06)	0.58
Calcium intake (mg/day)*					
1st quartile (338.2, -230.7–393.4)	39/290 (13.4%)	1.00		1.00	
2nd quartile (436.3, 393.5–479.8)	29/291 (10.0%)	0.71 (0.42, 1.18)	0.19	0.66 (0.38, 1.13)	0.13
3rd quartile (521.0, 479.9–584.4)	40/290 (13.8%)	1.03 (0.64, 1.66)	0.90	0.92 (0.55, 1.52)	0.73
4th quartile (667.7, 584.5–1478.5)	24/291 (8.2%)	0.58 (0.33, 0.98)	0.05	0.53 (0.30, 0.94)	0.03
*P* for trend		0.11		0.07	

*Quartile medians and range adjusted for energy intake by the residual method are given in parentheses.

^†^Adjusted for age, region of residence, smoking status, toothbrushing frequency, use of interdental brush, household income, and educational level.

Statistical power calculation revealed that, using our sample size, we could detect an association between calcium intake and periodontal disease for an OR of 0.518 with an accuracy of more than 80%.

No significant differences between never and ever smokers were observed in the association of calcium intake with the prevalence of periodontal disease (*P* =0.22, 0.40, and 0.98 for homogeneity of OR in the second, third, and highest quartiles, respectively).

## Discussion

In this study, we found that a higher intake of calcium was independently associated with a lower prevalence of periodontal disease among young Japanese women. Our results were in partial agreement with those from the Third National Health and Nutrition Examination Survey, which showed a significant inverse dose–response relationship between calcium intake as assessed by means of a 24-hour dietary recall and periodontal disease [8], but our results were inconsistent with those of a German study that showed no association between calcium intake as assessed by means of a seven-day food record and periodontal disease [12].

A US cross-sectional study reported that an inverse correlation between dietary calcium intake as assessed by means of a 24-hour dietary recall and periodontal index as assessed based on visual and radiographic data was of borderline significance (*r* = -0.24, *P* <0.075), while serum calcium concentration was not significantly correlated with periodontal index (*r* =0.16) [6]. These findings are in partial agreement with our results. Nevertheless, it should be noted that the above-mentioned studies used different definitions of outcome, study populations, exposure assessment methods, and confounding factors, thus limiting the feasibility of inter-study comparisons. In particular, the difference in average calcium intake between Japanese and Western populations should be taken into account when interpreting our results. According to the National Health and Nutrition Survey in Japan, the average daily per capita intake of calcium was 512 mg [19]. In contrast, for US women in 1999–2004, the average daily per capita intake of calcium was 756 mg [20]. The results of the present study suggest that, even at the relatively low levels habitually consumed in the Japanese population, calcium might have beneficial effects on periodontal disease.

Periodontal disease is characterized by the loss of tooth-supporting structures. In particular, the loss of alveolar bone is one of the most important hallmarks of periodontal disease. Calcium intake influences bone mineral density [21]. A cross-sectional study among young Japanese women showed a positive association between calcium intake and bone mineral density [22]. Increased intake of calcium might prevent bone loss because calcium suppresses the secretion of parathyroid hormone which leads to bone resorption [21].

Our study had certain methodological strengths. Study subjects were homogeneous in gender and age group. We were also able to control for relevant confounding factors. It is possible, however, that our results remain confounded by other potentially important factors, such as patterns of dental visits and alcohol consumption.

Several limitations should also be considered. The current study design was cross-sectional, and therefore the temporal nature of the association between calcium intake and periodontal disease could not be examined. We could not estimate the participation rate because the exact number of eligible pregnant women who were provided with a set of leaflets explaining the KOMCHS, an application form, and a self-addressed and stamped return envelope by the 423 collaborating obstetric hospitals is not available. We were also not able to assess the differences between participants and non-participants because information on personal characteristics such as age and socioeconomic status among non-participants is not available. Our subjects were probably not a representative sample of Japanese women in the general population, however. In fact, educational levels in the current study population were higher than in the general population. According to the 2000 population census of Japan, the proportions of women aged 30 to 34 years in Fukuoka Prefecture with <13, 13–14, ≥15, and an unknown number of years of education were 52.0%, 31.5%, 11.8%, and 4.8%, respectively [23]. The corresponding figures for the current study were 20.6%, 33.2%, 46.2%, and 0.0%, respectively. In addition, the prevalence of periodontal disease in our study population (11.4%) appeared to be lower than that in the sample of women aged 30 to 34 years in the National Survey of Dental Diseases, conducted in 2011 (14.3%), in which periodontal disease was likewise defined as one or more periodontal sites with a PPD of 4.0 mm or deeper [24]. With regard to dietary intake, however, calcium intake in this study population (500 mg/day) is similar to that in the general population (512 mg/day) [19].

Our DHQ could only approximate consumption and was designed to assess dietary intake for one month prior to completing the questionnaire. We believe that the possibility of non-differential exposure misclassification would introduce a bias toward the null. In the present study, assessment of diet was performed during pregnancy. Substantial changes in diet in the previous month were experienced by 341 pregnant women because of nausea gravidarum (322 women), maternal and fetal health (18 women), and other reasons (1 woman). The results of a sensitivity analysis which excluded these 341 women were similar to those in the overall analysis: the adjusted OR between extreme quartiles was 0.51 (95% CI: 0.26–0.99, *P* for trend =0.14). In the present study, data on serum calcium concentrations were not available.

In the current study, oral examinations were performed by dental hygienists. The dental hygienists were given detailed criteria for performing the examinations, but they received no specific training aimed at standardizing the procedures. In addition, no reliability assessment of measurements was carried out in the present study. Therefore, it is unknown whether intra- and interexaminer consistency was established. Further, because partial mouth recording was used in the present study, the prevalence of periodontal disease may have been underestimated. Moreover, our case definition of periodontal disease was based solely on the measurement of PPD, that is, the distance from the gingival margin to the base of the gingival sulcus or periodontal pocket. Measurements of PPD and clinical attachment level correlate well in many groups, especially younger populations, and both are accepted as measures of periodontal status [25].

## Conclusions

Findings from this cross-sectional study among young adult Japanese women suggest that calcium intake may be associated with periodontal disease. Calcium intake might have beneficial effects on periodontal disease even at the relatively low levels of intake that are typical in Japan. Further studies are required to confirm these findings and to understand the mechanisms behind the observed association between calcium intake and periodontal disease.

## Abbreviations

CI: Confidence interval
CPI: Community periodontal index
DHQ: Diet history questionnaire
KOMCHS: Kyushu Okinawa Maternal and Child Health Study
OR: Odds ratio
PPD: Probing pocket depth.
